# Characterizing psychopharmacological prescribing practices in a large cohort of adolescents with borderline personality disorder

**DOI:** 10.1186/s40479-024-00262-3

**Published:** 2024-08-06

**Authors:** Sarah Hauryski, Alexandra Potts, Alison Swigart, Dara Babinski, Daniel A. Waschbusch, Lauren N. Forrest

**Affiliations:** 1 Department of Psychiatry and Behavioral Health, Penn State College of Medicine, Hershey, PA USA; 2 Department of Psychiatry, Medical University of South Carolina, Charleston, USA

**Keywords:** Adolescent psychiatry, Clinical pharmacology, Psychiatry, Behavioral health

## Abstract

**Background:**

Psychiatric medications are not efficacious for treating borderline personality disorder (BPD), yet many patients with BPD are prescribed multiple psychiatric medications. This study aimed to (1) characterize psychiatric medication prescribing practices in adolescents with BPD and (2) assess whether demographic features are associated with prescribing practices.

**Method:**

This sample was *N* = 2950 pediatric patients with BPD (ages 10–19) across the U.S. Data came from the NeuroBlu database, which includes data from 30 U.S. healthcare systems and hundreds of hospitals. Poisson regressions and chi-squared tests determined whether gender, race, and ethnicity were associated with (1) number of unique psychiatric medications prescribed and (2) number of unique medication classes prescribed.

**Results:**

Roughly two-thirds (64.85%) of youth were prescribed any medications. Of these youth, 79.40% were prescribed ≥ 2 unique medications and 72.66% were prescribed ≥ 2 unique medications classes. The mean number of unique medications was 3.50 (*SD* = 2.50). The mean number of unique medication classes was 2.35 (*SD* = 1.15). The most commonly prescribed medication classes were antidepressants and antipsychotics, which were often prescribed in combination. Poisson regressions showed that boys were prescribed more unique medications *(M* = 3.67) than girls (*M* = 3.47). Non-Latinx youth were prescribed significantly more unique medications (*M* = 44.12) than Latinx youth (*M* = 3.60, *p* = .01).

**Conclusions:**

Results characterize psychiatric medication prescribing practices in youth with BPD. Prescribing practices vary by demographics, such that boys and non-Latinx youth are prescribed more medications than girls and Latinx youth, respectively. These demographic differences suggest that prescribers may treat BPD differently based on patient demographic characteristics.

**Supplementary Information:**

The online version contains supplementary material available at 10.1186/s40479-024-00262-3.

## Introduction

Borderline personality disorder (BPD) is a psychiatric disorder characterized by emotion dysregulation, impulsivity, unstable interpersonal relationships, unstable sense of self, feelings of worthlessness, and repeated self-injurious behavior. While many consider BPD to be a disorder found primarily in adulthood, BPD symptoms often manifest in adolescence [16, 20, 24, 42, 45]. If adolescent-onset symptoms persist for more than a year, BPD can be diagnosed [19].

Regardless of whether BPD onsets in adolescence vs. adulthood, there are no medications shown to be effective in treating BPD [47], and there are no psychiatric medications approved by the U.S. Food and Drug Administration to treat BPD [36]. However, existing pharmacological treatment guidelines vary. The National Institute for Health and Clinical Excellence guidelines state that pharmacotherapy should not be used to treat BPD symptoms, but that pharmacotherapy could be considered for treating comorbid psychiatric disorders among patients with BPD [21, 30]. The American Psychiatric Association guidelines state that BPD symptom-targeted pharmacotherapy could be used in conjunction with psychotherapy, which is the first-line treatment [7].

Despite pharmacotherapy for BPD being either not recommended at all or only in conjunction with psychotherapy, many people with BPD are prescribed psychiatric medications [5, 32]. The most commonly prescribed psychiatric medications are antidepressants, antipsychotics, mood stabilizers, and benzodiazepines [5, 21]. Although pharmacotherapy does not treat BPD’s underlying causes or mechanisms, pharmacotherapy may provide short-term symptom relief [32, 51] and may be prescribed to manage a crisis [7]. If pharmacotherapy is used, it is advised to prescribe only a single psychiatric medication at a time [4, 7].

In addition to the fact that pharmacological treatment in BPD is common despite no medications being effective for BPD specifically, many patients with BPD are often treated with *multiple* psychiatric medications [5, 32, 35]. This is referred to as polypharmacy [41, 45]. Polypharmacy can be quantified based on the number of medications or the number of medication *classes*. By either measure, polypharmacy is common in people with BPD. Studies find that 67–80% of patients with BPD are prescribed multiple medications [5, 32] and that two-thirds of patients (67%) are prescribed ≥ 2 medication classes [32]. Polypharmacy studies have been conducted among patients in psychiatric hospitals, outpatient psychiatric departments [5] and unspecified clinical sites [32]. Importantly, all studies, to our knowledge, have included adults only.

A clear gap in the BPD pharmacotherapy literature is that we are aware of only one study that has quantified the extent of pharmacotherapy overall, or polypharmacy specifically, in *adolescents* with BPD. A European Research Network Study on Borderline Personality Disorder found that 85% of youth had taken at least 1 psychotropic drug and that multiple prescriptions were more frequent than single prescriptions [6]. The limited knowledge on pharmacotherapy in youth with BPD may be due to the mismatch between DSM-5 and current clinical practice. Despite the DSM-5 allowing for BPD diagnoses prior to age 18 and the growing evidence and consensus for the validity and reliability of adolescent-onset BPD, some clinicians remain skeptical and reluctant to make a BPD diagnosis in adolescents [16, 24, 42].

Understanding the landscape of psychiatric prescribing practices for adolescents with BPD is important for multiple reasons. First, there is limited, if any, research on the long-term effects of psychopharmacological treatment in adolescents [39]. Second, valid concerns exist that psychiatric medications could have harmful effects on adolescents' rapidly developing brains [10]. Third, adolescents may be more vulnerable to adverse effects of psychotropic medications, and the risk–benefit ratio for medication in youth is often different than for adults [39].

The goal of the present study was to characterize psychiatric prescribing practices among adolescents with BPD. We had three aims. Aim 1 was to quantify the proportion of adolescents with BPD with versus without psychiatric medications prescribed between ages 10–19. Aim 2 was to characterize prescribing practices in adolescents with BPD, based on (1) the number of unique psychiatric medications prescribed between ages 10–19 and (2) the number of unique medication *classes* prescribed between ages 10–19. Aim 3 was to assess whether gender, race, or ethnicity were associated with prescriptions. We investigated the potential for demographic differences due to the underlying disparities in the healthcare system [27] and evidence that diagnosis of and treatment for psychiatric conditions varies based on race and ethnicity [9]. For instance, within a sample of 7.5 million patients, Native American/Alaskan Native patients had the highest rate of any psychiatric diagnosis (20.6%), while Asians received the lowest rates (7.5%). Further, amongst patients with a psychiatric diagnosis, non-Hispanic white patients were significantly more likely (77.8%) than other racial-ethnic groups to receive psychiatric medications [9].

## Method

### Participants

Data came from the NeuroBlu Database. NeuroBlu is a multisystem, multicenter server designed for behavioral health research. The version of data used in this analysis (22R5) includes data from 30 systems and hundreds of sites across the United States. All data were recorded as part of standard delivery of care. Data were obtained retroactively, and consent could not have been reasonably obtained [31]. NeuroBlu combined all sites’ de-identified data into a single cohesive, longitudinal data set. All data meet the Observational Health Data Sciences and Informatics and Observational Medical Outcomes Partnership data standards. IRB approval from Pennsylvania State University was obtained (STUDY00024964).

To obtain the sample used in analyses, we performed three steps. First, we selected all participants who had BPD diagnoses based on ICD-9 and/or ICD-10 codes. Then we determined the age of BPD diagnosis for each participant. This was calculated for each participant by taking the birth years and subtracting it from the year of first BPD diagnosis. Lastly, we selected only participants who were diagnosed with BPD between the ages of 10 and 19, inclusive, consistent with the World Health Organization’s definition of adolescence [50]. This resulted in a sample size of *N* = 2950. Moreover, our prescription data was limited to prescriptions that were prescribed between the ages of 10–19, to provide a clear picture of psychiatric medication prescribing practices for adolescents with BPD, during adolescence [50].

### Measures

All variables were derived from electronic medical records and listed in the NeuroBlu database. Variables included in the current study were gender, race, ethnicity, year of birth, year of BPD diagnosis, and psychiatric prescriptions.

The prescription data are not linked to a specific diagnosis. Prescriptions were categorized into medication classes by NeuroBlu and in consultation with our psychiatrist co-author (AS). However, not all medication classes were considered for analysis. We only considered medication classes that may be used to treat BPD [44] and medication classes that have previously been included in BPD polypharmacy studies [5, 21, 25, 28, 43]. The medication classes considered were anxiolytics, antidepressants, antipsychotics, anticonvulsants, and lithium. Lithium was considered its own class due to its unique characteristics and not fitting into any of the other classes. Supplemental Table 1 lists the specific medications that were considered within each medication class.

We include four prescription-related outcomes. The first outcome is whether patients had any psychiatric medications prescribed between ages 10–19. The remaining outcomes apply only to those with medications. The second outcome is being prescribed ≥ 2 medications between ages 10–19. The third outcome is the number of unique medications prescribed between ages 10–19. For example, if an individual had prescriptions for fluoxetine and sertraline, they would be counted as having two prescriptions. The fourth outcome is the number of unique medication *classes* prescribed between ages 10–19. Using the example above, this individual would be counted as having one medication class, as both fluoxetine and sertraline fall within the same medication class (antidepressants).

For the third outcome (i.e., number of unique medications), we present data for three time periods. The first time period is the total number of unique medications between ages 10–19. The second time period is medications that were first prescribed before BPD diagnosis. The third time period is medications that were first prescribed after BPD diagnosis. This dataset does not have a way to link the medication to a specific diagnosis, so differentiating medications prescribed before vs. after BPD diagnosis allows for understanding of any potential prescribing differences that may have arisen once BPD diagnoses were made.

Similarly, for the fourth outcome (i.e., number of medication classes), we present data for four time periods: medication classes that were (1) prescribed between ages 10–19 overall, (2) first prescribed before BPD diagnosis, (3) first prescribed on the same day the BPD diagnosis was made, and (4) first prescribed after BPD diagnosis.

### Data analytic plan

All analyses were performed in R [34]. Chi-square tests compared demographic characteristics based on (1) psychiatric medication status between ages 10–19 and (2) multiple medications vs. single medication. An independent samples t-test was used to compare age of first BPD diagnosis between those who were vs. were not prescribed medications. Descriptive statistics were calculated for the number of unique medications, the number of unique medication classes, the maximum number of medication classes prescribed between age 10–19, and the most common medication class combinations prescribed between ages 10–19. Poisson regressions determined whether gender, race, and ethnicity were associated with the number of unique medications.

In supplemental analyses, we stratified demographic characteristics by when medications were prescribed (prior to BPD diagnosis vs. after BPD diagnosis). We completed Poisson analyses to determine associations between demographic characteristics and numbers of medications and medication classes for each time period. The results of these analyses were similar to the main analyses and are thus presented in Supplemental Tables 2–6.

We additionally calculated the timing of psychiatric medications relative to BPD diagnosis (Supplemental Table 7) and the number of people who did vs. did not have comorbid psychiatric disorders stratified by psychiatric medication status (Supplemental Tables 8–9).

## Results

Table 1**s**hows the sample demographic characteristics for the sample. Most participants were girls (81.46%, *n* = 2403) and White (49.36%, *n* = 1456). Ethnicity was not reported for half the sample (50.64%, *n* = 1494); most of those with ethnicity data were not Latinx (34.24%, *n* = 1010).

**Table 1:** Demographic characteristics for patients with borderline personality disorder with and without psychiatric medications between ages 10–19

	Total Overall (*N* = 2950)		No Medications (*N* = 1038)		Any Medications (*N* = 1912)		≥ 2 medications (*N* = 1514)	
	*n*	%	*n*	%	*n*	%	*n*	%
Gender								
Girl	2403	81.46	814	33.87	1589	66.13	1256	79.04
Boy	544	18.44	224	41.18	320	58.82	257	80.31
Not reported	3	0.10	0	0.00	3	100.00	1	33.33
Race								
American Indian or Alaska Native	11	0.37	3	27.27	8	72.73	8	100.00
Asian	35	1.19	13	37.14	22	62.86	17	77.27
Black or African American	661	22.41	226	34.19	435	65.81	364	83.68
Multiracial	14	0.47	4	28.57	10	71.43	10	100.00
Native Hawaiian or Other Pacific Islander	82	2.78	13	15.85	69	84.15	51	73.91
White	1456	49.36	510	35.03	946	64.97	751	79.39
Asked but unknown	1	0.03	0	0.00	1	100.00	1	100.00
Not reported	690	23.39	269	38.99	421	61.01	312	74.11
Ethnicity								
Latinx	445	15.08	146	32.81	299	67.19	232	77.59
Not Latinx	1010	34.24	263	26.04	747	73.96	633	84.74
Asked but unknown	1	0.03	0	0.00	1	100.00	1	100.00
Not reported	1494	50.64	628	42.03	866	57.97	648	74.83

% for No Medications and Medication are % overall (out of total overall), whereas ≥ 2 medication % is out of number of people with medications

Ex. No medication % = 814/2403 = 33.87%; Any Medication % = 1589/2403 = 66.13%

Ex. ≥ 2 medications % = 1256/1589 = 79.04%

Table 1 shows demographic characteristics based on psychiatric medication status. Almost two-thirds of patients (64.81%, *n* = 1912) had at least one psychiatric medication between ages 10–19. Among all patients (i.e., regardless of medication status), over half (51.32%) were prescribed ≥ 2 medications between ages 10–19. However, among only patients with medications, 79.18% (*n* = 1514) were prescribed ≥ 2 psychiatric medications between ages 10–19.

More girls had any prescriptions compared to boys (χ^2^ = 10.05, *p* = 0.001). However, girls and boys had similar prevalence of being prescribed multiple medications (χ^2^ = 0.02, *p* = 0.90). For race, chi square tests were conducted only with White and Black participants, given the small sample sizes for all other races. The proportions of people who did vs. did not have prescriptions were similar for White and Black youth (χ^2^ = 0.11, *p* = 0.74). However, being prescribed multiple medications was descriptively higher in Black youth (83.68%) than in White youth (79.39%, χ^2^ = 0.32, *p* = 0.57). For ethnicity, the chi square included only individuals who were Latinx and not Latinx. Prescriptions were more common in non-Latinx youth vs. Latinx youth (χ^2^ = 6.67, *p* = 0.01). Similarly, being prescribed multiple medications was more common in non-Latinx youth vs. Latinx youth (84.74% vs 77.59%, χ^2^ = 6.67, *p* = 0.01). Age of first BPD diagnosis did not significantly differ between those with vs. without prescriptions (*t* = 0.11, *p* = 0.91).

### Number of unique medications

Table 2 shows descriptive statistics for the number of unique medications and medication classes prescribed between ages 10–19.

**Table 2:** Descriptive statistics for the number of (1) unique psychiatric medications and (2) unique psychiatric medication classes per patient between ages 10–19, before diagnosis, and after diagnosis

	*N*	*min*	*median*	*max*	*mean*	*sd*
Unique psychiatric medications						
Overall	1912	1	3	17	3.44	2.43
Before BPD diagnosis	1483	1	2	13	2.77	1.98
After BPD diagnosis	1372	1	2	16	2.81	1.97
Unique psychiatric medication classes						
Overall	1912	1	2	5	2.29	1.08
Before BPD diagnosis	1483	1	2	5	2.04	0.97
After BPD diagnosis	1372	1	2	5	2.06	1.03

*BPD* borderline personality disorder. The Before BPD diagnosis and After BPD diagnosis time periods are not mutually exclusive. That is, the Before BPD diagnosis time period shows descriptive statistics for the number of unique psychiatric medications prescribed before BPD was diagnosed (regardless of whether these patients also had psychiatric medications prescribed after BPD was diagnosed). Similarly, the After-BPD diagnosis time period shows descriptive statistics for the number of unique psychiatric medications prescribed after BPD was diagnosed (regardless of whether patients also had psychiatric medications prescribed before BPD was diagnosed)

Among patients with prescriptions (*N* = 1912), the range of unique medications was 1–17, with a mean of 3.44 (*SD* = 2.43) and median of 3 unique medications. The range of unique medication *classes* was 1–5, with a mean of 2.29 (*SD* = 1.08) and median of 2 unique medication classes. Table 2 also shows the range, mean, standard deviation, and median of unique medications overall and unique medication *classes* prescribed prior to BPD diagnosis vs. after BPD diagnosis.

Table 3 shows the number of unique medications between ages 10–19, stratified by gender, race, and ethnicity.

**Table 3:** Descriptive statistics and Poisson regression results comparing number of unique psychiatric medications between ages 10–19 by demographic characteristics

	*N*	mean	sd	median	*b*	exp(*b*)	se	*t*	*p*
Gender									
Girl (reference)	1589	3.42	2.41	3.0					
Boy	320	3.58	2,53	3.0	.04	1.05	.04	1.06	.29
Not reported	3	1.33	0.58	1.0					
Race									
American Indian or Alaska Native	8	3.38	1.30	3.0	-0.05	0.95	0.25	-0.19	.85
Asian	22	3.45	2.67	2.0	-0.02	0.98	0.15	-0.16	.87
Black or African American	435	3.70	2.31	3.0	0.04	1.05	0.04	1.12	.26
Multiracial	10	4.60	2.01	4.0	0.26	1.30	0.19	1.35	.18
Native Hawaiian or Other Pacific Islander	69	2.97	2.01	2.0	-0.18	0.84	0.09	-1.96	.06
White (reference)	946	3.54	2.57	3.0					
Asked but unknown	1	5.00	n/a	5.0	n/a				
Not reported	421	3.01	2.21	2.0	n/a				
Ethnicity									
Latinx	299	3.51	2.61	3.0	-0.1	0.87	0.05	-2.89	< .001
Not Latinx (reference)	747	4.03	2.66	3.0					
Asked but unknown	1	8.00	n/a	8.0	n/a				
Not reported	866	2.91	2.00	2.0	n/a				

Poisson Regression did not include “Asked but unknown” and “Not Reported” groups

Poisson regressions assessed whether each demographic characteristic was associated with the number of unique medications. For gender, there were not significant differences (*p* = 0.29), though at a descriptive level, boys were prescribed more unique medications than girls. Across racial groups, the number of medications prescribed was not significantly different (*p*s = 0.06–0.87). Multiracial youth had the highest number of unique medications (*M* = 4.60, *SD* = 2.01), followed by Black or African American youth (*M* = 3.70, *SD* = 2.31), while Native Hawaiian or Other Pacific Islander youth (*M* = 2.97, *SD* = 2.01) had the lowest number of unique medications. For ethnicity, non-Latinx adolescents were prescribed significantly more unique medications (*M* = 4.03, *SD* = 2.66) than Latinx adolescents (*M* = 3.51, *SD* = 2.61; *p* < 0.001).

### Medication classes

Table 4 shows the number of people with prescriptions for five psychiatric medication classes overall, and the medication classes stratified by whether the first medication within the class was prescribed (1) before BPD diagnosis, (2) on the same day as BPD diagnosis, or (3) following BPD diagnosis.

**Table 4:** Frequency of each medication class being prescribed overall, and frequencies of when psychiatric medication classes were first prescribed (i.e., prior to BPD being diagnosed, on the same day as BPD diagnosis, or after BPD was diagnosed) between ages 10–19

	Received medication class at any time between ages 10–19		First medication prescribed before BPD diagnosis		First medication prescribed the same day as BPD diagnosis		First medication prescribed after BPD diagnosis	
	*N*	% of *N* = 1912 with medications	*n*	% of *N* who received medication class	*n*	% of *N* who received medication class	*n*	% of *N* who received medication class
Anticonvulsants	731	38.23	301	41.18	176	24.08	254	34.75
Antidepressants	1493	78.09	679	45.48	377	25.25	437	29.27
Antipsychotics	1279	66.89	621	48.55	326	25.49	332	25.96
Anxiolytics	629	32.90	270	42.93	132	20.99	227	36.09
Lithium	253	13.23	92	36.36	56	22.13	105	41.50

The groups are mutually exclusive within medication classes. If a patient is prescribed multiple psychiatric medications of the same class, only the first prescription date of the psychiatric medication class is considered. Except where otherwise noted, percentages should be interpreted across the row. For example, for Anticonvulsant medication class precents sum across the row to equal 100 (41.18% + 24.08% + 34.75% = 100%)

These groups are mutually exclusive. For instance, consider a participant who had two prescriptions within the antidepressant class: sertraline and fluoxetine. Sertraline was prescribed three years prior to BPD diagnosis, while fluoxetine was prescribed one year following BPD diagnosis. This participant would be counted only as having their first antidepressant medication prescribed prior to BPD diagnosis. Overall, these results show when medication classes are most commonly and initially prescribed relative to the timing of BPD diagnosis.

Antidepressants and antipsychotics were prescribed most commonly overall and within each unique time period (before BPD, same day as BPD, after BPD). When investigating prescription timing for each medication class, anticonvulsants, antidepressants, antipsychotics, and anxiolytics were most often prescribed before BPD diagnosis, compared to on the same day as diagnosis or following diagnosis. Only lithium was prescribed more often after BPD diagnosis, compared to before BPD diagnosis or on the same day as BPD diagnosis. No medications were most often prescribed on the same day as BPD diagnosis compared to other time periods.

**Fig. 1:**
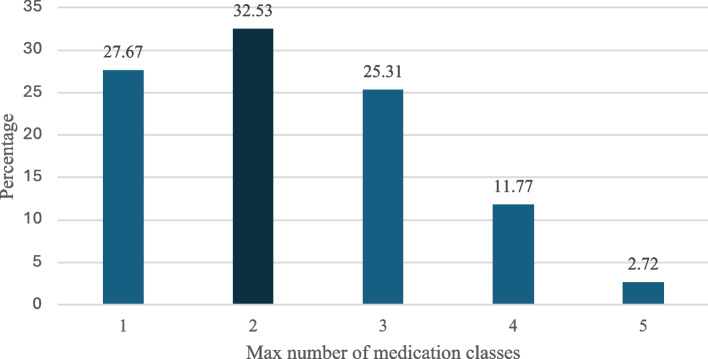
Distribution of participants based on the maximum number of medication classes prescribed between age 10–19

Figure 1 shows the distribution of participants based on the maximum number of medication classes they were prescribed between age 10–19. Roughly one-third of participants were prescribed a maximum of two unique medication classes (32.53%). Roughly one quarter of participants were prescribed only one medication class (27.67%) and another quarter were prescribed a maximum of three medication classes (25.31%).

**Fig. 2:**
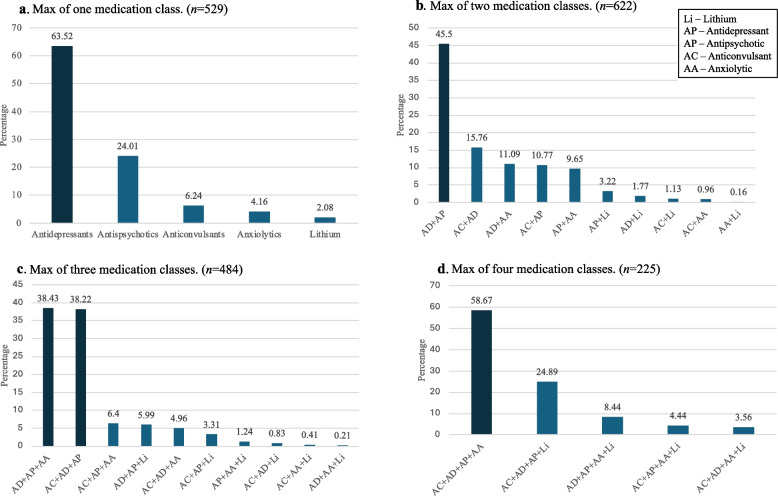
**a-d** Distributions of participants based on the maximum number of prescribed medication classes (1–4) between age 10–19

Figure 2 shows the most common medication classes and the most common combinations of medication classes prescribed between age 10–19, based on individuals’ maximum number of medication classes prescribed. For example, Fig. 2a shows that of participants who were prescribed a maximum of one medication class, almost two-thirds of prescriptions were within the antidepressant category (63.52%). Figure 2b shows that of participants who were prescribed a maximum of two medication classes, the most common medication combination was antidepressants + antipsychotics (45.50%). Figure 2c shows that of participants who were prescribed a maximum of three medication classes, the most common medication combinations were (1) antidepressants + antipsychotics + anxiolytics (38.43%) and (2) antidepressants + antipsychotics + anticonvulsants (38.22%). Finally, Fig. 2d shows that of participants who were prescribed a maximum of four medication classes, the most common medication combination was antidepressants + antipsychotics + anxiolytics + anticonvulsants (58.67%).

## Discussion

This study characterized the psychiatric prescribing practices among adolescents with BPD during adolescence (ages 10–19) and examined whether being prescribed any or multiple psychiatric medications was associated with gender, race, and/or ethnicity. We found that roughly two-thirds of adolescents with BPD were prescribed psychiatric medications. Of those who were prescribed psychiatric medications, roughly 80% were treated with *multiple* psychiatric medications. These findings are similar to results from a study of service utilization among adolescents with BPD from the European Research Network on BPD, which found that 85% had been taken at least 1 psychotropic drug, and that multiple prescriptions were more common than single prescriptions [6]. This proportion of adolescents with BPD who are prescribed multiple psychiatric medications is comparable to the proportion of adults with BPD who are prescribed multiple psychiatric medications [5]. However, youth with BPD were prescribed an average of 3.50 medications during adolescence, which is higher than the average number of 2–3 medications reported in adults [21, 25]. The average number of unique medication classes prescribed to youth (2.35) is nearly identical to the average prescribed to adults (2.4; [25]).

These results demonstrate that pharmacotherapy overall and being prescribed multiple medications specifically are very common in adolescents with BPD, despite pharmacotherapy not having substantial empirical backing for BPD at any age, [5, 32, 35] let alone for adolescents. We also found that more girls with BPD were prescribed medications than boys with BPD. Latinx adolescents with BPD were prescribed fewer medications than non-Latinx adolescents with BPD.

### Specific medication prescriptions relative to timing of BPD diagnosis

The most commonly prescribed psychiatric medication classes in youth with BPD were antidepressants and antipsychotics, and many of the most medication class combinations included both antidepressants and antipsychotics. This is similar to the most commonly prescribed medication classes in adults with BPD [5, 6]. Antidepressant, antipsychotics, anticonvulsants, and anxiolytic medications were most often prescribed prior to BPD diagnosis. Antidepressants being commonly prescribed prior to BPD diagnosis is not surprising, given that antidepressants are the most commonly prescribed medication class among youth with many different psychiatric diagnoses (with the exception of attention-deficit/hyperactivity disorder and externalizing disorders; [23]). The finding that antipsychotics were commonly prescribed to adolescents diagnosed with BPD and that they were most commonly prescribed prior to BPD diagnosis, was surprising, given the significant risk profile of antipsychotics [39]. Antipsychotics confer risk of long-term serious side effects, including irreversible movement disorders, weight gain, hyperlipidemia, metabolic syndrome, diabetes, and elevated prolactin levels [39]. Similarly, anticonvulsant medications have a broad range of impairing side effects, including irritability, aggression, hyperactivity, headache, gastrointestinal distress, drowsiness, dizziness, and blurred vision, as well as potentially serious side effects such as hepatic failure, pancreatitis, hyponatremia, and hematologic abnormalities [15, 26]. These potential adverse effects are particularly important to consider for adolescents, given that adolescents are going through periods of rapid hormonal and neurological development [39].

Lithium prescriptions were most common after BPD diagnosis. There are a handful of possible explanations for this finding. First, lithium may have been most often prescribed following BPD diagnosis because medications with fewer possible side effects and not requiring blood monitoring were initially prescribed but did not result in adequate treatment response and therefore more intensive medications were prescribed. Second, people with BPD often experience suicidal thoughts and behaviors, and are at high risk for death by suicide [29]. Providers may be more focused on a patient’s risk for suicidal thoughts and behaviors upon a patient receiving a BPD diagnosis and may consider starting lithium because it is one of the few medications that reduces long-term suicide risk [14]. However, we believe it is important to reiterate two points. First, our data do not provide a way to link prescriptions to diagnoses, so we cannot draw a conclusion as to whether medications were prescribed for BPD symptoms specifically or for a comorbid condition. Second, although there are understandable reasons providers may use pharmacotherapy as a treatment for BPD symptoms, there are no medications shown to be effective in treating BPD pathology [47].

### Demographic differences

We found some evidence for prescribing differences based on gender, ethnicity, and race. First, medications overall were more common in girls versus boys with BPD, but *multiple* medications were similar in girls and boys with BPD. This was somewhat surprising, given that polypharmacy in general is more prevalent in men versus women, and this gender difference has become widespread in child and adolescent psychiatry [17]. However, it is worth noting that women are much more likely than men to be represented in BPD treatment-seeking samples [38], despite population-based surveys generally finding that BPD prevalence is similar across genders [38]. Thus, pharmacotherapy data for boys or men with BPD is more limited [12] and this pattern of findings requires replication.

Second, we found evidence for ethnic differences in the extent of pharmacotherapy, where Latinx adolescents were prescribed fewer medications compared to non-Latinx adolescents. However, Black adolescents with BPD were prescribed descriptively *more* medications compared to White adolescents, though this result was not statistically significant. Research describing racial and ethnic differences in pharmacotherapy for BPD is limited. However, some racial and ethnic differences have been noted for other mental health conditions, which together may suggest disparate mental health treatment among racial and ethnic minority groups [8]. For example, one study noted that Black and Latinx patients were less likely to be prescribed an antidepressant when compared to White patients [8]. Another study investigated racial and ethnic differences in (1) antidepressant medications among youth with depression and (2) antipsychotic medications among youth with neurodevelopmental disorders. For both diagnostic groups, racial and ethnic minority youth were less likely than White youth to receive pharmacotherapy [23].

Even though racial and ethnic minorities’ lower extents of psychiatric medications in the context of BPD could be seen as a more favorable outcome, given that polypharmacy for BPD is generally not recommended, these lower rates could also be an indicator of racial bias and racism within the US healthcare system [22]. Indeed, when laypeople and mental health providers are presented with the exact same vignettes of people experiencing psychopathology, they rate that psychopathology is *less* distressing to Black people versus White people [18]. Moreover, when clinicians believe a patient’s distress is lower, they are more likely to provide less intensive treatment [18]. Although we are not aware of data assessing whether these Black-White differences extend to Latinx people, this is an important direction for future study.

Another possible explanation for racial and ethnic minority adolescents’ lower extents of any psychiatric medications could be due to differences in cultural beliefs about healthcare [3]. For example, Hispanic patients are more likely to attribute mental health concerns to family conflict, lack of a support system, and religious circumstances than to a medical condition requiring medical care [40]. If patients attribute their symptoms to an external cause that a medication would not impact, they may be less likely to accept a medication as treatment.

However, there are several important caveats to the demographic differences reported here. First, it is possible that there are demographic differences in BPD diagnosis—i.e., some groups may be more likely to receive a BPD diagnosis, while other groups may be less likely to receive a BPD diagnosis [13, 49]. Our dataset does not capture those with false negative or false positive diagnoses. Second, it is also possible that there are demographic differences in the people with BPD who do vs. do not seek or receive treatment for BPD. For instance, girls and women with eating disorders are more likely to seek eating disorder treatment than boys and men with eating disorders [11, 48]. Given that our dataset only includes people being treated for BPD, we cannot assess how demographic characteristics may or may not relate to treatment status.

### Clinical implications

These findings may inform prescribing practices for youth with BPD in several ways. First, results increase awareness of the near ubiquity of psychopharmacological treatment among youth with BPD. Specifically, nearly two-thirds of adolescents with BPD are being treated with psychiatric medications between ages 10–19 and more than 80% of medicated youth are being treated with multiple psychiatric medications, *despite no medications being effective for treating BPD overall* [47] and little research, to our knowledge, that has investigated medication efficacy in youth with BPD. Second, the demographic differences highlight the possibility of disparate treatment being provided for the same diagnosis based on the individual’s gender, race or ethnicity. This highlights the need to address health disparities for the treatment of BPD across racial and ethnic groups of adolescents with BPD.

### Strengths, limitations, and future directions

Strengths of this study include a large sample size and a sample of adolescents with BPD, who are a vulnerable but previously unstudied population for characterizing psychopharmacological prescribing practices. Given the substantial growth and development during adolescence, studying prescribing practices overall and by drug class for adolescents with BPD fills an important gap in the literature.

Limitations are as follows. First, these data do not allow us to link prescriptions to a specific diagnosis, so we are not able to discern whether medications were prescribed to treat BPD symptoms versus comorbid psychiatric disorders. It makes intuitive sense that the more comorbid diagnoses someone has, the more medications they may be prescribed. Although these data do not allow us to definitively assess this intuition, Supplemental Table 9 shows that the number of medications prescribed do not systematically differ based on the presence or absence of comorbidities. Further, given that comorbidity is nearly ubiquitous among people with BPD [13], it is possible that some medications were prescribed for treating symptoms other than those of BPD. However, we did limit our included medications to those most commonly used to treat BPD symptoms specifically. This approach has pros and cons. On the one hand, we included medications based on the existing literature (which exclusively included adult samples), and it therefore reflects a narrow estimate of psychiatric prescribing practicing during adolescence for youth with BPD. On the other hand, we excluded stimulants, which is a frequently prescribed psychiatric medication class for youth with externalizing and neurodevelopmental disorders, such as oppositional defiant disorder and attention-deficit/hyperactivity disorder [1, 33]. There is accumulating evidence suggesting that externalizing problems and neurodevelopmental disorders should also be considered in the etiology and treatment of BPD in adolescents [2, 46]. Therefore, stimulants may need to be considered in future studies assessing psychiatric prescribing practices in adolescents with BPD. Second, these data do not provide a way to determine whether medications were prescribed concurrently, and, as such, we do not refer to our findings as indicators of polypharmacy per se, given that polypharmacy typically implies multiple medications prescribed simultaneously. Third, the dataset does not contain data on features that may be associated with prescribing practices, such as states or regions, visit types, or prescriber credentials. Fourth, these data could not determine whether patients were receiving other forms of psychological treatment, such as psychotherapy. Some parents or patients may not have had access to psychotherapy, and psychiatric medication may have been more accessible. Fourth, the data do not allow assessing the efficacy or impact of psychiatric medications on symptom severity, though we again highlight the widespread consensus that psychiatric medications are not recommended or evidence-based treatment for BPD [5, 32].

Given these limitations, a pressing future direction to explore relates to a common and difficult ethical dilemma: is some treatment better than no treatment? If we assume that psychiatric medications are not effective for youth with BPD (as is the case for adults with BPD; [36, 47]), but youth with BPD cannot access more evidence-based treatments like psychotherapy [6], what impact do psychiatric medications have on youth’s longer-term functioning and healthcare costs associated with BPD? In other words, if gold-standard treatments are not available or accessible [6], and yet adolescents are experiencing distressing, impairing, and potentially life-threatening BPD symptoms, are psychiatric medications more helpful or more harmful, and does the risk–benefit profile differ for short-term vs. long-term use? We believe this issue requires more research, including perspectives from multiple stakeholders, including patients, their families, and prescribers.

Another important future direction of research is assessing differences in prescribing patterns for people with BPD based on gender, race, and ethnicity, and specifically research on bias and discrimination that people from marginalized groups experience in healthcare settings [22]. Relatedly, assessing prescribing practices and differences based on sexual orientation is also important, considering that (1) sexual minority individuals are far more likely to be diagnosed with BPD compared to heterosexual individuals but (2) several BPD criteria could be viewed as normative responses to living in a structurally stigmatizing environment [37].

## Conclusion

In summary, we found that approximately two-thirds of adolescents with BPD had at least one psychiatric medication. Of youth with BPD who had any psychiatric medications, 79.40% were prescribed ≥ 2 medications, with an average of 3.50 different medications and 2.35 medication classes. Findings also showed demographic differences that may suggest differential treatment practices for racial and ethnic minority youth with BPD. Future research is needed that investigates the efficacy of pharmacotherapy for youth with BPD when first-line treatments (i.e., psychotherapy) are not available. Overall, characterizing prescribing practices in youth with BPD extends the field’s understanding of the scope of psychiatric prescribing practices in young people with BPD.

### Supplementary Information

Supplementary Material 1.

## Data Availability

No datasets were generated or analysed during the current study.
