# Supplementary material for: Characterizing psychopharmacological prescribing practices in a large cohort of adolescents with borderline personality disorder
Source: Borderline Personal Disord Emot Dysregul. 2024 Aug 6;11:17. doi: 10.1186/s40479-024-00262-3 (PMC11302085; doi:10.1186/s40479-024-00262-3)
Supplement: Supplementary file 1 — Supplementary Material 1. [file 40479_2024_262_MOESM1_ESM.docx]

| **Drug Class** | **Drugs** |
| --- | --- |
| Anxiolytics | meprobamate, diazepam, oxazepam, buspirone, hydroxyzine, alprazolam, clorazepate, lorazepam, halazepam, chlordiazepoxide, kava root, bromazepam, prazepam, clobazam, potassium clorazepate (oral), etizolam (oral) |
| Antidepressants | tranylcypromine, trazodone, trimipramine, amitriptyline, amoxapine, nefazodone, duloxetine, escitalopram, desipramine, desvenlafaxine, nortriptyline, paroxetine, mirtazapine, phenelzine, doxepin, sertraline, venlafaxine, bupropion, fluvoxamine, protriptyline, fluoxetine, imipramine, isocarboxazid, ketamine, maprotiline, citalopram, clomipramine, esketamine, tryptophan, mianserin, viloxazine, moclobemide, dothiepin, butriptyline, milnacipran, reboxetine, melitracen, vilazodone, hyperici herba (systemic), levomilnacipran, vortioxetine, agomelatine |
| Antipsychotics | thioridazine, thiothixene, mesoridazine, paliperidone, trifluoperazine, molindone, ziprasidone, perphenazine, risperidone, droperidol, pimozide, lithium carbonate, prochlorperazine, fluphenazine, aripiprazole, haloperidol, quetiapine, olanzapine, loxapine, chlorpromazine, clozapine, reserpine, deserpidine, Butaperazine, trifluperidol, zuclopenthixol, benperidol, iloperidone, acepromazine, bromperidol, promazine, periciazine, amisulpride, metylperon, pipamperone, chlorprothixene, perazine, pipothiazine, sulpiride, fluanisone (oral), aripiprazole, lauroxil, cariprazine, lumateperone, asenapine, lurasidone, pimavanserin, brexpiprazole |
| Anticonvulsants | mephenytoin, lamotrigine, levetiracetam, fosphenytoin, tiagabine, oxcarbazepine, phenobarbital, pregabalin, carbamazepine, phenytoin, topiramate, zonisamide, valproate, ethosuximide, primidone, methsuximide, felbamate, gabapentin, clonazepam, cannabidiol, metharbital, trimethadione, rufinamide, valproic acid, vigabatrin, dipropylacetamide, lacosamide, aminobutyric acid, brivaracetam, cenobamate, perampanel, eslicarbazepine |
| Lithium | lithium carbonate, lithium citrate, lithium |

**Supplemental Table 1.** Psychiatric Medication Classification

| Demographics | *N* | mean | sd | median | *b* (est) | exp(*b*) | *se* | *t* | *p* |
| --- | --- | --- | --- | --- | --- | --- | --- | --- | --- |
| Gender | | | | | | | | | |
| Girl (reference) | 1232 | 2.75 | 1.93 | 2.0 |  |  |  |  |  |
| Boy | 248 | 2.90 | 2.19 | 2.0 | 0.05 | 1.06 | 0.05 | 1.11 | .27 |
| Not reported | 3 | 1.33 | 0.58 | 1.0 | n/a |  |  |  |  |
| Race | | | | |  |  |  |  |  |
| American Indian or Alaska Native | 6 | 2.33 | 1.03 | 2.0 | -0.17 | 0.84 | 0.32 | -0.54 | .59 |
| Asian | 18 | 3.33 | 2.74 | 2.0 | 0.19 | 1.20 | 0.16 | 1.19 | .24 |
| Black or African American | 347 | 3.15 | 2.09 | 3.0 | 0.13 | 1.14 | 0.04 | 2.88 | < .001 |
| Multiracial | 9 | 3.56 | 1.24 | 3.0 | 0.25 | 1.28 | 0.21 | 1.18 | .24 |
| Native Hawaiian or Other Pacific  Islander | 54 | 2.43 | 1.64 | 2.0 | -0.13 | 0.88 | 0.11 | -1.24 | 0.22 |
| White (reference) | 740 | 2.77 | 2.01 | 2.0 |  |  |  |  |  |
| Asked but unknown | 1 | 1.00 | n/a | 1.0 | n/a |  |  |  |  |
| Not reported | 308 | 2.36 | 1.71 | 2.0 | n/a |  |  |  |  |
| Ethnicity | | | | |  |  |  |  |  |
| Latinx | 227 | 2.70 | 2.14 | 2.0 | -0.18 | 0.83 | 0.06 | -3.20 | <.01 |
| Not Latinx (reference) | 589 | 3.23 | 2.12 | 3.0 |  |  |  |  |  |
| Asked but unknown | 1 | 7.00 | n/a | 7.0 | n/a |  |  |  |  |
| Not reported | 666 | 2.38 | 1.67 | 2.0 | n/a |  |  |  |  |
| *Note:* Poisson Regression did not include “Asked but unknown” and “Not Reported” groups | | | | | | | | |  |

**Supplemental Table 2.** Descriptive statistics and Poisson regression results comparing number of unique psychiatric ***medications*** prescribed ***before*** borderline personality disorder diagnosis by demographic characteristics.

**Supplemental Table 3.** Descriptive statistics and Poisson regression results comparing number of unique psychiatric ***medications*** prescribed ***after*** BPD diagnosis by demographic characteristics.

| Demographics | *N* | mean | sd | median | *b* (est) | exp(*b*) | *se* | *t* | *p* |
| --- | --- | --- | --- | --- | --- | --- | --- | --- | --- |
| Gender | | | | | | | | | |
| Girl (reference) | 1135 | 2.79 | 1.96 | 2.0 |  |  |  |  |  |
| Boy | 237 | 2.95 | 2.03 | 2.0 | 0.05 | 1.06 | 0.05 | 1.14 | .26 |
| Not reported | 0 | n/a | n/a | n/a |  |  |  |  |  |
| Race | | | | |  |  |  |  |  |
| American Indian or Alaska Native | 7 | 3.00 | 1.53 | 3.0 | 0.04 | 1.04 | 0.26 | 0.15 | .88 |
| Asian | 12 | 2.58 | 1.38 | 2.0 | -0.11 | 0.90 | 0.21 | -0.52 | .60 |
| Black or African American | 324 | 2.94 | 1.81 | 2.0 | 0.02 | 1.02 | 0.05 | 0.42 | .67 |
| Multiracial | 6 | 4.00 | 2.76 | 3.0 | 0.32 | 1.38 | 0.24 | 1.47 | .17 |
| Native Hawaiian or Other Pacific  Islander | 51 | 2.14 | 1.39 | 1.0 | -0.30 | 0.74 | 0.11 | -2.61 | .01 |
| White (reference) | 677 | 2.88 | 2.10 | 2.0 |  |  |  |  |  |
| Asked but unknown | 1 | 4.00 | NA | 3.0 | n/a |  |  |  |  |
| Not reported | 294 | 2.60 | 1.93 | 2.0 | n/a |  |  |  |  |
| Ethnicity | | | | |  |  |  |  |  |
| Latinx | 230 | 2.94 | 2.09 | 2.0 | -0.08 | 0.92 | 0.06 | -1.52 | .13 |
| Not Latinx (reference) | 540 | 3.20 | 2.18 | 3.0 |  |  |  |  |  |
| Asked but unknown | 1 | 7.00 | NA | 7.0 | n/a |  |  |  |  |
| Not reported | 601 | 2.41 | 1.63 | 2.0 | n/a |  |  |  |  |

*Note:* Poisson Regression did not include “Asked but unknown” and “Not Reported” groups

**Supplemental Table 4.** Descriptive statistics and Poisson regression results comparing number of unique psychiatric medication ***classes*** by demographic characteristics.

| Demographics | *N* | mean | sd | median | *b* (est) | exp(*b*) | *se* | *t* | *p* |
| --- | --- | --- | --- | --- | --- | --- | --- | --- | --- |
| Gender | | | | | | | | | |
| Girl (reference) | 1589 | 2.29 | 1.08 | 2.0 |  |  |  |  |  |
| Boy | 320 | 2.34 | 1.06 | 2.0 | 0.03 | 1.02 | 0.03 | 0.78 | .44 |
| Not reported | 3 | 1.33 | 0.58 | 1.0 |  |  |  |  |  |
| Race | | | | |  |  |  |  |  |
| American Indian or Alaska Native | 8 | 2.75 | 1.16 | 2.0 | 0.17 | 1.18 | 0.15 | 1.11 | .27 |
| Asian | 22 | 2.18 | 0.96 | 2.0 | -0.06 | 0.94 | 0.10 | -0.61 | .54 |
| Black or African American | 435 | 2.42 | 1.05 | 2.0 | 0.04 | 1.04 | 0.03 | 1.56 | .12 |
| Multiracial | 10 | 3.10 | 0.88 | 3.0 | 0.29 | 1.33 | 0.13 | 2.25 | .02 |
| Native Hawaiian or Other Pacific Islander | 69 | 2.09 | 0.94 | 2.0 | -0.11 | 0.90 | 0.06 | -1.77 | .08 |
| White (reference) | 946 | 2.32 | 1.11 | 2.0 |  |  |  |  |  |
| Asked but unknown | 1 | 3.00 | n/a | 3.0 | n/a |  |  |  |  |
| Not reported | 421 | 2.10 | 1.02 | 2.0 | n/a |  |  |  |  |
| Ethnicity | | | | |  |  |  |  |  |
| Latinx | 299 | 2.22 | 1.03 | 2.0 | -0.4 | 0.87 | 0.03 | -4.44 | <.01 |
| Not Latinx (reference) | 747 | 2.56 | 1.13 | 3.0 |  |  |  |  |  |
| Asked but unknown | 1 | 5.00 | n/a | 5.0 | n/a |  |  |  |  |
| Not reported | 865 | 2.09 | 0.99 | 2.0 | n/a |  |  |  |  |

*Note:* Poisson Regression did not include “Asked but unknown” and “Not Reported” groups

**Supplemental Table 5.** Descriptive statistics and Poisson regression results comparing number of unique psychiatric medication ***classes*** prescribed ***before*** borderline personality disorder diagnosis by demographic characteristics

| Demographics | *N* | mean | sd | median | *b* (est) | exp(*b*) | *se* | *t* | *p* |
| --- | --- | --- | --- | --- | --- | --- | --- | --- | --- |
| Gender | | | | | | | | | |
| Girl (reference) | 1232 | 2.04 | 0.96 | 2.0 |  |  |  |  |  |
| Boy | 248 | 2.06 | 0.98 | 2.0 | 0.01 | 1.01 | 0.03 | 0.21 | .83 |
| Not reported | 3 | 1.33 | 0.58 | 1.0 |  |  |  |  |  |
| Race | | | | |  |  |  |  |  |
| American Indian or Alaska Native | 6 | 1.83 | 0.41 | 2.0 | -0.11 | 0.90 | 0.21 | -0.53 | .60 |
| Asian | 18 | 2.11 | 1.02 | 2.0 | 0.03 | 1.03 | 0.11 | 0.29 | .77 |
| Black or African American | 347 | 2.23 | 0.99 | 2.0 | 0.09 | 1.09 | 0.03 | 2.89 | <.01 |
| Multiracial | 9 | 2.56 | 0.73 | 2.0 | 0.22 | 1.25 | 0.14 | 1.56 | .12 |
| Native Hawaiian or Other Pacific  Islander | 54 | 1.85 | 0.74 | 2.0 | -0.10 | 0.91 | 0.07 | -1.41 | .16 |
| White (reference) | 740 | 2.04 | 1.00 | 2.0 |  |  |  |  |  |
| Asked but unknown | 1 | 1.00 | n/a | 1.0 | n/a |  |  |  |  |
| Not reported | 308 | 1.85 | 0.85 | 2.0 | n/a |  |  |  |  |
| Ethnicity | | | | |  |  |  |  |  |
| Latinx | 227 | 1.92 | 0.90 | 2.0 | -0.17 | 0.84 | 0.04 | -4.72 | <.01 |
| Not Latinx (reference) | 589 | 2.28 | 1.02 | 2.0 |  |  |  |  |  |
| Asked but unknown | 1 | 5.00 | n/a | 5.0 |  |  |  |  |  |
| Not reported | 666 | 1.97 | 0.89 | 2.0 |  |  |  |  |  |

*Note:* Poisson Regression did not include “Asked but unknown” and “Not Reported” groups

**Supplemental Table 6.** Descriptive statistics and Poisson regression results comparing number of unique psychiatric medication ***classes*** prescribed ***after*** borderline personality disorder diagnosis by demographic characteristics

| Demographics | *N* | mean | sd | median | *b* (est) | exp(*b*) | *se* | *t* | *p* |
| --- | --- | --- | --- | --- | --- | --- | --- | --- | --- |
| Gender | | | | | | | | | |
| Girl (reference) | 1135 | 2.04 | 1.04 | 2 | 0.03 | 1.03 | 0.04 | 0.88 | .38 |
| Boy | 237 | 2.11 | 0.99 | 2 |  |  |  |  |  |
| Not reported | 0 | n/a | n/a | n/a |  |  |  |  |  |
| Race | | | | |  |  |  |  |  |
| American Indian or Alaska Native | 7 | 2.57 | 1.27 | 3.0 | 0.21 | 1.23 | 0.17 | 1.24 | .22 |
| Asian | 12 | 1.83 | 0.83 | 2.0 | -0.12 | 0.88 | 0.15 | -0.84 | .40 |
| Black or African American | 324 | 2.16 | 0.99 | 2.0 | 0.04 | 1.04 | 0.03 | 1.13 | .26 |
| Multiracial | 6 | 2.67 | 1.37 | 3.0 | 0.25 | 1.28 | 0.18 | 1.37 | 17 |
| Native Hawaiian or Other Pacific Islander | 51 | 1.69 | 0.88 | 1.0 | -0.21 | 0.81 | 0.08 | -2.67 | .01 |
| White (reference) | 677 | 2.08 | 1.06 | 2.0 |  |  |  |  |  |
| Asked but unknown | 1 | 3.00 | n/a | 3.0 | n/a |  |  |  |  |
| Not reported | 294 | 1.92 | 0.99 | 2.0 | n/a |  |  |  |  |
| Ethnicity | | | | |  |  |  |  |  |
| Latinx | 230 | 2.07 | 1.01 | 2.0 | -0.08 | 0.92 | 0.04 | -2.10 | .04 |
| Not Latinx (reference) | 540 | 2.24 | 1.08 | 2.0 |  |  |  |  |  |
| Asked but unknown | 1 | 5.00 | n/a | 5.0 | n/a |  |  |  |  |
| Not reported | 601 | 1.88 | 0.95 | 2.0 | n/a |  |  |  |  |

*Note:* Poisson Regression did not include “Asked but unknown” and “Not Reported” groups

**Supplemental Table 7.** Time (in months) between when psychiatric medication classes were prescribed relative to borderline personality disorder diagnosis

|  | Received medication class at any time between ages 10–19 | | First medication prescribed before BPD diagnosis | | | | | First medication prescribed after BPD diagnosis | | | | |
| --- | --- | --- | --- | --- | --- | --- | --- | --- | --- | --- | --- | --- |
|  | *N* | % of *N* = 1912 with medications | *n* | % of *N* who received medication class | median | mean | sd | *n* | % of *N* who received medication class | median | mean | sd |
| Anticonvulsants | 731 | 38.23 | 301 | 41.18 | 4.40 | 12.00 | 16.59 | 254 | 34.75 | 2.33 | 6.56 | 9.87 |
| Antidepressants | 1493 | 78.09 | 679 | 45.48 | 5.09 | 12.12 | 17.27 | 437 | 29.27 | 0.72 | 3.94 | 8.46 |
| Antipsychotics | 1279 | 66.89 | 621 | 48.55 | 4.93 | 11.89 | 17.15 | 332 | 25.96 | 1.08 | 5.09 | 9.18 |
| Anxiolytics | 629 | 32.90 | 270 | 42.93 | 2.25 | 6.83 | 10.80 | 227 | 36.09 | 2.07 | 6.42 | 10.76 |
| Lithium | 253 | 13.23 | 92 | 36.36 | 3.58 | 9.36 | 12.69 | 105 | 41.50 | 2.63 | 7.61 | 11.47 |

*Note.* BPD = borderline personality disorder.

*Note:* Roughly 20-25% of patients had a given medication class prescribed on the same day as their BPD diagnosis. Those individuals are not included in this table, as the median, mean, and SD are all 0.

| Comorbid diagnosis | Total (%) (*N*=2950) | No medications (*n*=1038) | Any medications (*n*=1912) |
| --- | --- | --- | --- |
| Yes | 2669 (90.47) | 942 (90.75) | 1727 (90.32) |
| No | 281 (9.53) | 96 (9.25) | 185 (9.68) |

**Supplemental Table 8.**

*Note.* Comorbid diagnoses are only those that were diagnosed prior to diagnosis of borderline personality disorder.

**Supplemental Table 9.** Number of unique medications stratified by demographics, separated by whether or not people had comorbid diagnoses diagnosed prior to BPD diagnosis, among *N* = 1912 individuals with medications prescribed between ages 10–19

|  | *N* | | Mean | | | | SD | | | | Median | | | | |
| --- | --- | --- | --- | --- | --- | --- | --- | --- | --- | --- | --- | --- | --- | --- | --- |
| Comorbidities | No  *n* = 185 | Yes  *n* = 1727 | No | | Yes | | No | | Yes | | No | | Yes | | |
| Gender |  |  |  | |  | |  | |  | |  | |  | | |
| Girl | 158 | 1431 | 3.33 | | 3.43 | | 2.34 | | 2.41 | | 3 | | 3 | | |
| Boy | 26 | 294 | 2.88 | | 3.64 | | 1.75 | | 2.58 | | 2 | | 3 | | |
| Not reported | 1 | 2 | 2.0 | | 1.0 | | n/a | | 0 | | 2 | | 1 | | |
| Race | | | | | | | | | | | | | | |  |
| American Indian or Alaska Native | 1 | 7 | 3.0 | 3.43 | | n/a | | 1.40 | | 3 | | 3 | |  |  |
| Asian | 1 | 21 | 2.0 | 3.52 | | n/a | | 2.71 | | 2 | | 2 | |  |  |
| Black or African American | 49 | 386 | 3.39 | 3.74 | | 1.60 | | 2.39 | | 3 | | 3 | |  |  |
| Multiracial | 2 | 8 | 5.0 | 4.50 | | 1.41 | | 2.20 | | 5 | | 4 | |  |  |
| Native Hawaiian or Other Pacific Islander | 1 | 68 | 3.0 | 2.97 | | n/a | | 2.02 | | 3 | | 2 | |  |  |
| White | 96 | 850 | 3.33 | 3.56 | | 2.71 | | 2.56 | | 3 | | 3 | |  |  |
| Asked but unknown | 0 | 1 | n/a | 5.0 | | n/a | | n/a | | n/a | | 5 | |  |  |
| Not reported | 35 | 386 | 2.83 | 3.03 | | 1.76 | | 2.25 | | 2 | | 2 | |  |  |
| Ethnicity | | | | | | | | | | | | | | |  |
| Latinx | 29 | 270 | 3.24 | 3.54 | | 2.43 | | 2.63 | | 3 | | 3 | |  |  |
| Not Latinx | 55 | 692 | 3.58 | 4.07 | | 2.57 | | 2.66 | | 3 | | 3 | |  |  |
| Asked but unknown | 1 | 0 | 8 | n/a | | n/a | | n/a | | 8 | | n/a | |  |  |
| Not reported | 100 | 765 | 3.04 | 2.89 | | 1.98 | | 2.00 | | 3 | | 2 | |  |  |
